# Ratios and determinants of maternal mortality: a comparison of geographic differences in the northern and southern regions of Cameroon

**DOI:** 10.1186/s12884-020-02879-y

**Published:** 2020-03-31

**Authors:** Catherine Meh, Amardeep Thind, Amanda L. Terry

**Affiliations:** 1 Department of Epidemiology and Biostatistics, Western University, 1151 Richmond St., London, ON N6A 5C1 Canada; 2 Department of Family Medicine, Western University, 1151 Richmond St., London, ON N6A 5C1 Canada; 3 Schulich Interfaculty Program in Public Health, Schulich School of Medicine and Dentistry, Western University, 1151 Richmond St., London, ON N6A 5C1 Canada

**Keywords:** Cameroon, Maternal mortality, Maternal health

## Abstract

**Background:**

While maternal mortality has declined worldwide in the past 25 years, this is not the case for Cameroon. Since there is a predominantly young population in this country, high maternal mortality ratios may persist. Maternal mortality ratios vary within countries, yet it is unknown if the North and South, the most distinct parts of Cameroon, differ in terms of ratios and determinants of maternal mortality.

**Methods:**

This study explored ratios and determinants of maternal mortality in women of childbearing age (15–49 years) and assessed differences between the North and South. We used the Cameroon Demographic and Health Surveys (2004 and 2011) to extract a sample of 18,665 living or deceased women who had given birth. Multivariable logistic regression was used to explore the relationship between maternal mortality and sociocultural, economic and healthcare factors.

**Results:**

Maternal mortality ratios were different for the two regions and increased in the North in 2011 compared to 2004. In the North, any level of education and being Muslim were protective against maternal mortality. Meanwhile, the odds of maternal mortality decreased with increasing age, and having secondary or higher education in the South. Domestic violence and ethnicity were associated with maternal death in the South. Increasing parity was protective of maternal death in both the North and South.

**Conclusions:**

Maternal mortality ratios and determinants varied between women of childbearing age in the North and South of Cameroon. These reinforce recommendations for region specific strategies that will improve health communication, community education programs, curb domestic violence and train more community health workers to connect pregnant women with the health system. Programs to reduce maternal death among women with low parity and little or no education should be national priority.

## Background

Every day, just over 800 maternal deaths occur worldwide, affecting women of childbearing age (15–49 years of age) in mainly resource-limited settings [1]. Maternal mortality is an indicator of women’s status and inequality and reflects a country’s development in relation to its health system [2, 3]. Consequences of maternal mortality go beyond the death of an expectant mother. It is a tragedy for herself, her child, family and community.

Several factors at individual and community level are linked directly or indirectly with maternal mortality. Direct factors comprise pregnancy complications such as hemorrhage, eclampsia, sepsis, abortion, and obstructed labor [3, 4]. Indirect factors include pre-existing conditions like malaria, anemia and nutrition which are aggravated by pregnancy [4]. Age and parity, which are linked to a woman’s reproductive status, are associated with maternal death. Access to health services, women’s health seeking behavior and use of health services can indirectly lead to maternal death. Socioeconomic, environmental, and cultural factors impact maternal mortality [3, 5].

Millennium Development Goal (MDG) 5 brought a new international focus to this issue [6, 7]. Globally, efforts have led to approximately 44% decline in maternal deaths [1]. Sustainable Development Goal (SDG) 3 seeks to further reduce global maternal mortality ratios (MMR) to less than 70 per 100,000 live births [1] and to less than 140 per 100,000 live births in high burden countries by 2030 [8]. Variations in MMRs and its determinants exist both between and within countries, challenging maternal health programs.

In 2011, Cameroon had a maternal mortality ratio of 782 per 100,000 live births as compared to 669 /100,000 in 2004 and 454/100,000 in 1998 [9–12]. Cameroon did not meet MDG 5 target of reducing maternal mortality by 75% in 2015 and is challenged by the even more ambitious SDG 3. With a predominantly young population, approximately 60% of whom are under 25 years of age, a high fertility rate of 5.1 children per woman, and one-third of women having their first child before 18 years [10], high MMRs may likely persist. For Cameroon to get on track with SDG 3, maternal mortality needs to fall at least below 140/100,000 live births. To achieve this target, the country has started to focus on interventions like family planning [10], infection control [11–13], increased training of health professionals [13–15] and financial support for these programs.

The few studies that address determinants of maternal mortality in Cameroon are health facility-based or have small community-based samples [16]. Age, parity, socioeconomic status, no antenatal care, having been referred from smaller health centers for pregnancy and birth complications, past preterm births and grand multiparity arise as determinants from these studies [17].

Cameroon is broadly bisected by the Adamawa Plateau, north of which are predominantly Fulani (Peuhl) and Kirdi people, and to the south are largely Highlanders and Bantu people [18]. The northern area (North), a savanna and steppe (Sudano-Sahel) zone, has a hot and dry tropical climate, a large Muslim population, and is home to some of Cameroon’s poorest. The southern area (South), the more metropolitan part of the country, includes the western highlands and southern forest zones that are populated mainly by Christians. Such disparities can influence MMRs and its determinants; there is, however, little research to shed light on this issue.

The rising MMR points to a growing problem with the overall health status of women and the health system in this country. Assessing MMRs and determinants of maternal mortality in these regions can highlight disparities, allowing policymakers to target appropriate evidence-based interventions. Hence, this study aimed to explore MMRs and determinants of maternal mortality among women of childbearing age (15–49 years) in the North and South of Cameroon.

## Methods

**Fig. 1:**
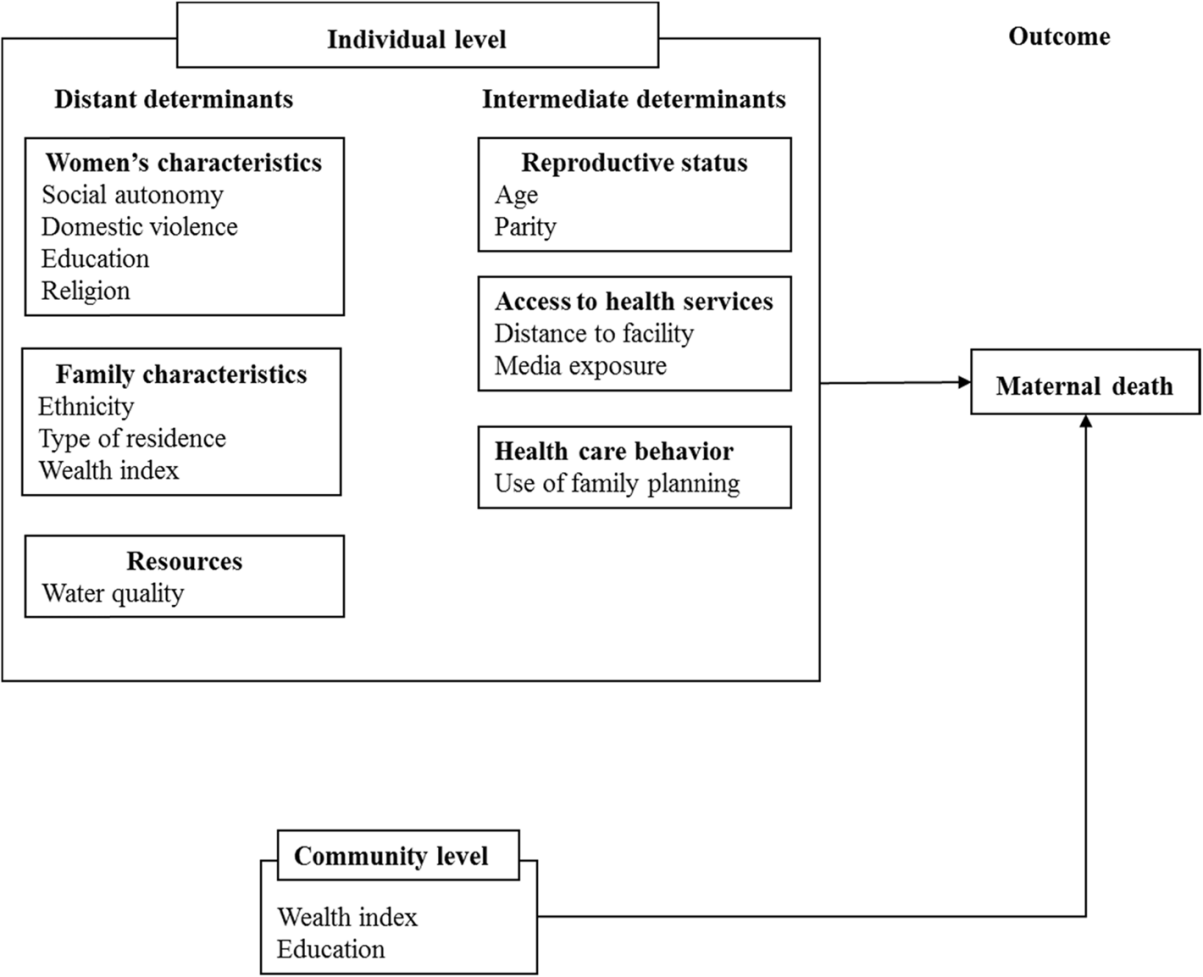
Adapted Framework (McCarthy & Maine) Determinants of Maternal Mortality

Our study used a modified McCarthy and Maine’s framework (Fig. 1) to analyze distant, intermediate and proxy determinants of maternal mortality in the North and South of Cameroon [3]. Intermediate determinants such as age and parity, directly influence a woman’s likelihood of dying. Meanwhile, distant determinants like education, indirectly act through the intermediate factors. This framework also distinguishes between individual and community level determinants.

Cameroon Demographic and Health Surveys (CDHS) of 2004 and 2011 were used for this study. CDHS 2018 data are not yet available for analysis. These are nationally representative household surveys for population, health, and nutrition indicators [19] conducted every 5 to 7 years in several Low and Middle Income Countries in partnership with the United States Agency for International Development (USAID) [20]. CDHS data of 2004 and 2011 were analyzed separately to estimate MMR for the North and South. They were then pooled to create the final study sample to assess determinants of maternal mortality.

This was a cross-sectional study to assess determinants of maternal mortality in women of childbearing age (15–49 years). CDHS uses the direct sisterhood method [21] (a variant of the sisterhood method [22]) to identify deceased siblings (among which are cases of maternal mortality) through selected questions. Participants in the CDHS included a total of 26,082 (10,656 from 2004 and 15,426 from 2011) women of childbearing age 15–49 years. Women who had been pregnant and given birth to at least one child were included. Participants consisted of study respondents (living women) and their deceased siblings who were identified through the direct sisterhood method and had died of pregnancy related factors.

A respondent was eligible for inclusion in this study if her total number of children ever born was one or greater. Deceased women were included in this study as cases of maternal death when they were identified through a respondent (sibling born of the same mother) as having died while pregnant, during childbirth or within 6 weeks after giving birth. Only age and parity were reported for deceased siblings. Other characteristics were ascribed from their living siblings (respondents). These included ethnicity, religion, type of residence, educational level, wealth, type of contraception use, media exposure, distance to health facility, water quality, social autonomy, attitude towards domestic violence, and location. We assumed the deceased siblings were of the same household and thus would share similar characteristics [23–25].

### Variables

Maternal death was the outcome variable for this study. Respondents were asked whether a female sibling died while pregnant, during childbirth, or within 6 weeks or 2 months of giving birth. Respondents could indicate yes, no or don’t know. This was then dichotomized; cases of maternal mortality (deceased siblings) were coded as 1 and living women (respondents) were coded as 0.

Data were extracted on age (in years), and parity (number of children ever born). These were treated as continuous variables. Other variables included distance to health facility which was self-reported into four categories - “no problem”, “small problem”, “big problem”, and “unknown”. For media exposure, a composite variable was created for frequency of listening to the radio, watching television, and reading a newspaper or magazine. These were categorized as no exposure (0), low exposure (1–3), medium exposure (4–6) and high exposure (7–9). Method of contraceptive used had three categories: “no method”, “folk/traditional”, and “modern”.

Educational level was defined as “no education”, “primary”, and “secondary/higher”. Religion was grouped as “Catholics”, “other Christians”, “Muslims” and “Animist/none/other”. Respondents’ stated ethnicities were categorized into six major groups. Two variables for ethnicity were created and used for the separate regional analyses. This was to capture the varying ethnic composition of the North and South since region and ethnicity were correlated. Type of residence was dichotomized as urban or rural. Wealth index was measured with an asset score by CDHS and was characterized as poor, middle and rich. The source of water was classified as improved or unimproved based on whether it was protected from outside contamination by its natural construction or a deliberate intervention.

A composite variable was created for social autonomy using four measures of participation in decision making: woman’s involvement in decisions on her own health care, daily and large household purchases, and family/relative visits. The values for this composite variable ranged from 0 to 4, where 0 was no participation and 4 was for participation in decision making in all four individual measures.

Likewise, a composite variable for domestic violence was created. It measured a participant’s attitude towards husband/partner beating wife in four situations: for going out without telling husband/partner, neglecting children, arguing with husband/partner and refusing sex. They could respond “no” coded as 0 or “yes/don’t know” coded as 1. The response options for the composite variable were a sum of the individual scores ranging from 0 to 4. A score of 0 meant a woman did not approve of violence in any of the situations and a score of 4 was approval of violence in all four situations.

Responses for education and wealth index were aggregated at regional level to create community level variables. Categories for education were coded as follows: individuals with no education or with primary education were assigned a value of 0. Those who had secondary or higher education were assigned a value of 1. A new variable was then generated where the mean value was assigned to all individuals according to their region. This mean value represented the proportion of individuals with secondary or higher education in each region. Likewise, for community wealth, a value of 0 was assigned to individuals whose wealth index was poor or middle and those who were rich were coded as 1. The mean of these values was then assigned to individuals in the respective regions. This value represented the proportion of rich women in each region.

The 10 administrative regions and 2 major cities (Douala and Yaoundé) in Cameroon were the response options for the variable region. The 3 northernmost regions (Adamawa, North and Far North) were classed as North and the rest (East, South, Center, Littoral, West, North West, South West, Douala and Yaoundé) were grouped as South. Survey year was also included with 2004 and 2011 as its response categories.

### Missing data

Social autonomy and distance to facility data were not available in 2232 (11.9%) and 5764 (30.9%) of our overall sample. Predictive mean matching imputation [26] was performed for social autonomy where a mean value, derived from a set of observed values in the dataset (closest predicted mean), was imputed. Individuals with missing values for distance to health facility were categorized as unknown.

### Statistical analyses

All statistical analyses were performed using Stata SE13 [27]. These analyses were carried out on weighted data to adjust for sampling design, non-responsiveness, stratification and clustering. Maternal mortality ratios for 2004 and 2011 were computed for each region (North and South). Survey adjusted simple and multivariable logistic regression analyses were used to investigate the association of the independent variables and maternal mortality in the North and South of Cameroon. Statistical significance for all regressions performed was determined at *p* < 0.05.

## Results

### Descriptive analysis

**Fig. 2:**
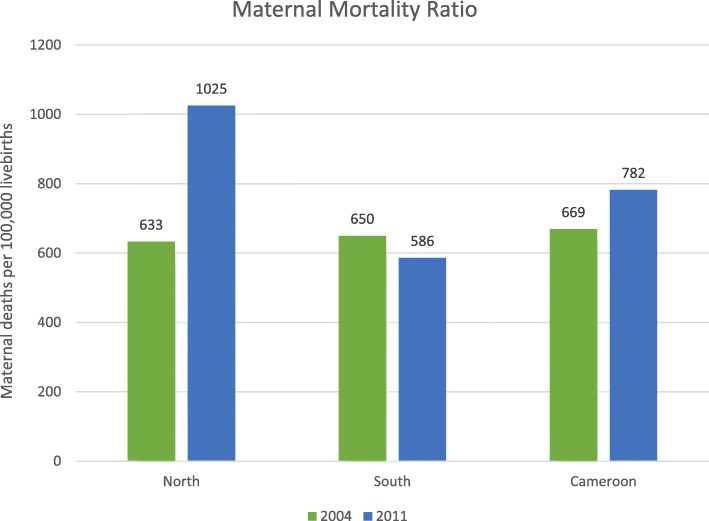
Maternal Mortality Ratio in the North and South Regions and Cameroon Overall in 2004 & 2011. Ratios for Cameroon obtained from CDHS Report for 2004 and 2011 [9, 28]

There were 571 (3.1%) maternal deaths, 204 (35.7%) of which were reported in CDHS 2004 for the period 1998–2004 and 367 (64.3%) for the period 2004–2011 in CDHS 2011. A majority of 357 (62.5%) death were reported in the South region. MMR for the North was 633/100,000 live births in 2004 and 1025/100,000 live births in 2011 (Fig. 2). For the South, MMR was 650/100,000 live births in 2004 and 586/100,000 live births in 2011 (Fig. 2).

The study sample consisted of 18,665 (18,094 living and 571 deceased) women of childbearing age (Table 1). Mean age was 30.8 years with most participants in the 20–24 and 25–29-year age groups. Average number of children was 3.8. For the North, there were 5610 women with a mean age of 30 years and an average of 4.4 children. A total of 13,055 women in the South had an average age of 31.2 years with 3.5 children.

**Table 1:** Characteristics of Women – North and South

Variable	North (*n* = 5610)	South (*n* = 13,055)
	n (%)	n (%)
**Survival status**		
Alive	5396 (96.2)	12,698 (97.3)
Dead	214 (3.8)	357 (2.73)
	**Mean (SD)**	**Mean (SD)**
**Community level**		
Community education (range: 0–1)	0.07 (0.26)	0.47 (0.5)
Community wealth (range: 0–1)	0.16 (0.36)	0.49 (0.5)
**Individual level**		
Age (range: 15–49)	30.0 (8.7)	31.2 (8.8)
Parity (range: 0–20)	4.4 (2.9)	3.5 (2.4)
Social autonomy (range: 0–4)	1.42 (SE 0.05)	2.35 (SE 0.02)
Domestic violence (range: 0–4)	1.38 (1.57)	1.13 (1.31)
**Categorical variables**	**N (%)**	**N (%)**
**Distance to facility**		
No Problem	1055 (18.81)	3452 (26.44)
small problem	929 (16.56)	2276 (17.43)
Big problem	1, 864 (33.2)	3325 (25.5)
unknown	1762 (31.4)	4002 (30.7)
**Media exposure**		
No exposure	3133 (55.9)	2498 (19.1)
Low exposure	1707 (30.4)	4934 (37.8)
Medium exposure	627 (11.2)	4196 (32.2)
High exposure	140 (2.5)	1423 (10.9)
**Contraception type**		
No method	5252 (93.7)	8624 (66.1)
Folk/traditional	90 (1.6)	2001 (15.3)
Modern	266 (4.7)	2430 (18.6)
**Education**		
No education	3609 (64.3)	733 (5.6)
Primary	1606 (28.6)	6188 (47.4)
Secondary/higher	395 (7.0)	6134 (46.9)
**Religion**		
Catholics	1018 (18.2)	5782 (44.4)
Other Christians	1236 (22.1)	5583 (42.8)
Muslims	2662 (47.6)	1009 (7.7)
Animist/None/Other	673 (12.0)	657 (5.0)
**Ethnicity**		
Arab-Choa/Peulh/Haoussa/Kanuri	1477 (26.4)	263 (2.0)
Biu-Mandara	1892 (33.8)	69 (0.5)
Adamaoua-Oubangui	1803 (32.2)	335 (2.6)
Bantoid south-west/Grassfields	47 (0.8)	2556 (19.6)
Bamilike/Bamoun/Côtier/Ngoe/Oroko	80 (1.4)	4511 (34.8)
Beti/Bassa/Mbam/Kako/Meka/Pygmé	72 (1.3)	4900 (37.7)
Stranger/other	224 (4.0)	377 (2.9)
**Type of residence**		
Urban	1601 (28.5)	6825 (52.3)
Rural	4009 (71.5)	6230 (47.7)
**Wealth index**		
Poor	3769 (67.2)	3516 (26.9)
Middle	972 (17.3)	3127 (23.9)
Rich	869 (15.49)	6412 (49.1)
**Water quality**		
Improved	2524 (46.2)	8283 (67.2)
Unimproved	2939 (53.8)	4035 (32.8)

### Bivariate analyses

In the North, odds of maternal death reduced with increasing age and parity. Women who reported having a big problem with distance to health facilities had increased odds of maternal mortality. Having primary education reduced the odds of maternal death by 37% for women in the North as compared to women without education.

In the South, odds of maternal death decreased with increasing age and parity. Increasing levels of domestic violence increased the odds of maternal death by 15%. Among the southern ethnic groups, the Beti/Bassa/Mbam/Kako/Meka/Pygmé ethnic group had higher odds of maternal death as compared to Bantoid southwest/Grassfield. For women in the South, living in a rural area increased the odds of maternal mortality by 30% (Table 2).

**Table 2:** Bivariate Analyses of Determinants of Maternal Mortality - North and South

Variable	North (*n* = 5610) OR (95% CI)	South (*n* = 13,055) OR (95% CI)
**Community level**		
Community education	**0.02 (0.001–0.47)**	0.99 (0.47–2.09)
Community wealth	**0.10 (0.02–0.64)**	0.74 (0.45–1.24)
**Individual level**		
Age	**0.92 (0.89–0.94)**	**0.94 (0.92–0.95)**
Parity	**0.69 (0.63–0.77)**	**0.73 (0.65–0.81)**
Social autonomy	1.02 (0.93–1.12)	0.96 (0.87–1.07)
Domestic violence	0.95 (0.86–1.05)	**1.15 (1.05–1.26)**
**Distance to facility**		
No problem	1	1
Small problem	1.49 (0.86–2.60)	0.80 (0.54–1.18)
Big problem	**1.88 (1.16–3.05)**	1.01 (0.72–1.41)
Unknown	**2.44 (1.51–3.93)**	1.02 (0.73–1.41)
**Media exposure**		
No exposure	1	1
Low exposure	0.83 (0.59–1.15)	0.80 (0.54–1.18)
Medium exposure	0.53 (0.28–1.00)	1.01 (0.73–1.53)
High exposure	0.64 (0.19–2.10)	1.02 (0.58–1.48)
**Contraception type**		
No method	1	1
Folk/traditional	1.56 (0.48–5.12)	0.94 (0.67–1.30)
Modern	0.71 (0.30–1.66)	0.95 (0.69–1.31)
**Education**		
No education	1	1
Primary	**0.63 (0.43–0.92)**	1.08 (0.63–1.87)
Secondary/higher	0.55 (0.26–1.15)	0.84 (0.49–1.45)
**Religion**		
Catholics	1.00	1
Other Christians	0.74 (0.45–1.21)	1.07 (0.82–1.41)
Muslims	0.69 (0.47–1.02)	1.03 (0.66–1.60)
Animist/None/Other	0.80 (0.47–1.36)	0.71 (0.39–1.29)
**Ethnicity**		
Arab-Choa/Peulh/Haoussa/Kanuri	1	–
Biu-Mandara	1.17 (0.82–1.66)	–
Adamaoua-Oubangui	1.23 (0.83–1.80)	–
Bantoid South-west/Grassfields	–	1
Bamilike/Bamoun/Côtier/Ngoe/Oroko	–	0.89 (0.61–1.28)
Beti/Bassa/Mbam/Kako/Meka/Pygmé	–	**1.55 (1.11–2.16)**
Stranger/Other	0.99 (0.53–1.89)	1.33 (0.82–2.17)
**Type of residence**		
Urban	1	1
rural	1.26 (0.93–1.71)	**1.30 (1.01–1.67)**
**Wealth index**		
Poor	1	1
Middle	0.72 (0.46–1.15)	1.06 (0.75–1.49)
Rich	0.59 (0.34–1.01)	0.80 (0.59–1.10)
**Water quality**		
Improved	1	1.00
Unimproved	1.21 (0.86–1.69)	1.11 (0.85–1.45)
**Year**		
2004	1	1
2011	1.80 (1.30–2.49)	0.98 (0.76–1.27)

**Bold:** statistical significance *p* < 0.05

**Ethnicity**: Two separate variables for ethnic composition were used for regional analyses

### Multivariable analyses

In the North, odds of maternal death were reduced by 29% with increasing parity. Primary or secondary/higher education significantly reduced the odds of maternal mortality as compared to no education. Being Muslim was protective against maternal mortality as compared to Catholics. Compared to 2004, odds of maternal mortality significantly increased in 2011 (Table 3).

**Table 3:** Multivariable Analyses of Determinants of Maternal Mortality: North and South

Variable	North (*n* = 5610) AOR (95% CI)	South (*n* = 13,055) AOR (95% CI)
**Community level**		
Community education	–	2.49 (0.42–14.84)
Community wealth	–	0.45 (0.15–1.41)
**Individual**		
Age	0.97 (0.95–1.00)	**0.97 (0.95–0.99)**
Parity	**0.71 (0.64–0.79)**	**0.74 (0.64–0.86)**
Social autonomy	1.10 (0.98–1.23)	1.11 (1.00–1.22)
Domestic violence	0.97 (0.87–1.09)	**1.15 (1.04–1.26)**
**Distance to facility**		
No problem	1	1
Small problem	0.84 (0.38–1.89)	0.66 (0.37–1.19)
Big problem	1.38 (0.76–2.53)	0.85 (0.55–1.29)
Unknown	1.32 (0.62–2.81)	0.86 (0.49–1.50)
**Media exposure**		
No exposure	1	1
Low exposure	0.97 (0.69–1.37)	0.94 (0.67–1.33)
Medium exposure	0.62 (0.28–1.36)	1.14 (0.76–1.71)
High exposure	1.13 (0.31–4.13)	1.00 (0.58–1.75)
**Contraception type**		
No method	1	1
Folk/traditional	2.37 (0.71–7.87)	1.18 (0.83–1.68)
Modern	1.19 (0.46–3.11)	1.04 (0.74–1.47)
**Education**		
No education	1	1
Primary	**0.41 (0.28–0.61)**	0.82 (0.43–1.57)
Secondary/higher	**0.25 (0.09–0.66)**	**0.48 (0.25–0.94)**
**Religion**		
Catholics	1	1
Other Christians	0.72 (0.44–1.20)	1.10 (0.82–1.48)
Muslims	**0.63 (0.40–0.99)**	0.95 (0.55–1.64)
Animist/None/Other	0.71 (0.39–1.29)	0.89 (0.48–1.68)
**Ethnicity**		
Arab-Choa/Peulh/Haoussa/Kanuri	1	–
Biu-Mandara	0.97 (0.63–1.47)	–
Adamaoua-Oubangui	1.21 (0.76–1.91)	–
Bantoid South-west/Grassfields	–	1
Bamilike/Bamoun/Côtier/Ngoe/Oroko	–	1.21 (0.78–1.87)
Beti/Bassa/Mbam/Kako/Meka/Pygmé	–	**1.80 (1.16–2.80)**
Stranger/other	1.04 (0.52–2.07)	1.65 (0.94–2.88)
**Type of residence**		
Urban	1	1
rural	0.71 (0.38–1.33)	1.19 (0.84–1.68)
**Wealth index**		
Poor	1	1
Middle	0.85 (0.40–1.79)	1.13 (0.80–1.59)
Rich	0.82 (0.32–2.13)	0.94 (0.59–1.49)
**Water quality**		
Improved	1	1
Unimproved	1.26 (0.87–1.84)	0.91 (0.66–1.24)
**Year**		
2004	**1**	1
2011	**2.15 (1.18–3.91)**	1.28 (0.80–2.06)

**Bold:** statistical significance p < 0.05

**Community level:** Not explored in the North because there were only three administrative regions

**Ethnicity**: Two separate variables for ethnic composition were used for regional analyses

Like the North, parity and education were also significant in the South, where the odds of maternal mortality decreased by 26% with increased parity and by 52% with a secondary/higher education. Conversely, age, domestic violence, and ethnicity were only significant in the South where odds of maternal death decreased by 3% with increasing age. An increase in the score for domestic violence by one unit increased the odds of maternal mortality by 15%, while members of the Beti/Bassa/Mbam/Kako/Meka/Pygmé ethnic group had almost twice the odds of maternal mortality as compared to Bantoid South-west/Grassfields (Table 3).

## Discussion

There were differences between the North and South in levels of material mortality. These differences were most visible in 2011 where maternal deaths increased in the North and slightly decreased in the South. The North and South also differed in terms of determinants of maternal mortality. In the North, there were significant associations between increasing parity, level of education, religion and reduction of maternal mortality while in the South, increasing age and parity, and education, reduced the odds of maternal mortality. Domestic violence and ethnicity were significantly associated with maternal mortality.

### Maternal mortality ratios in the north and south

The MMR for the North rose in 2011, which was a year with a significant increase in the odds of maternal death. This may explain the overall rise in national MMRs in Cameroon. The worsening poverty of residents in the North between 2001 to 2007 and again in 2014 [29], and the influx of large numbers of displaced persons between 2004 and 2011 [30] from conflict regions in neighboring countries could explain the spike in MMR in this region. Displaced women tend to be most affected and are especially vulnerable to poor health outcomes from lack of resources. This places an additional strain on already limited resources in the North which is characterized by a high proportion of women living in rural areas (about 70%), who are poor (over 60%) with a severe shortage of health workers in understaffed health facilities [31].

### Determinants of maternal mortality in the north and south

#### North

The greater risk of complications in women with no previous births may explain the high odds of maternal death for low parity. This is in line with findings from Bangladesh and other studies in Cameroon where nulliparity increased the risk of maternal mortality [32–34]. The cultural preference of many children explains the high fertility rate in Cameroon.

Lower education and low socioeconomic status may adversely impact employment and maternal health services’ utilization and outcomes [35]. This is pertinent in Cameroon where costs of health care are mostly borne by individuals. About 50% of maternal deaths in Cameroon were among women with minimal income [34].

The influence of traditional and cultural factors, especially on maternal health seeking behavior may be especially strong as seen in other studies [36, 37]. Even though primary education was decreed free in 2000/2001 in Cameroon [38], the high proportion of women with no education (over 60% in the North), may be an indication of the deep rooted nature of traditional and cultural values. Low rates of higher education may explain the higher maternal mortality in the region.

Given the influence of religious beliefs on health seeking behavior and the impact this has on health status (such as the presence of infectious diseases like HIV), this finding may be hinting at disparities in the health seeking behaviors of women of different religious groups in the North. For instance, more unsafe sexual behavior and a higher prevalence of HIV was reported in Cameroon among Christian than Muslim men [39]. These pose a health risk for wives/partners and may explain the lower odds of maternal mortality among Muslim women compared to their Catholic counterparts. Muslim communities prohibit certain behavior and practices such as the consumption of alcohol [40]. Alcohol is linked with maternal death through more frequent induced abortions, which are predominantly performed in illicit settings [41]. Religion, however, had little influence on Christian or Muslim women’s uptake of maternal health services in Nigeria, suggesting that other social conditions may explain this finding and should be explored [42].

#### South

There are inconsistent findings where a greater risk of maternal death has been shown among older age groups compared to younger age groups [33, 37, 43]; others have found no associations with age [44]. Factors such as early marriage (in Cameroon young girls can legally marry at 15 years), and early childbearing increase the proportion of adolescent pregnancies which are at higher risk for maternal mortality [45]. Primary education alone in the South (where almost 50% of women have secondary or higher education) does not make a significant difference in a woman’s odds for maternal mortality. Sexual violence was common among host and refugee populations in the South [46]. Intimate partner violence increases the risk of induced abortion [47]. While abortion remains illegal in Cameroon, women turn to illicit abortion that contribute to 9.6% of maternal deaths in sub-Saharan Africa [48]. Also, excessive consumption of alcohol in Cameroon can impact the frequency and severity of violence against women by their partners [49]. Domestic violence may persist in part due to societal norms that view it as a private issue between partners.

Unique practices within ethnic groups may impact maternal health. The Baya ethnic group used sugar cane peel to cut the umbilical cord during childbirth, indicating unhealthy obstetric practices among traditional birth attendants [50]. This ethnic group reported also more illnesses, favored early childbearing compared to others and preferred traditional to skilled birth attendants during childbirth [51].

As part of the main intervention (family planning) adopted to reduce maternal mortality in Cameroon, the impact of modern contraceptive use remains to be seen as uptake has been weak [10]. Though some elements (costs, availability and need for spousal/parental approval) may hinder uptake of contraceptives in women, media messages alone will not effectively influence use of contraceptives.

### Policy recommendations

The North is plagued with severe shortages of health care providers and provides refuge to displaced persons from conflict zones. The government of Cameroon is committed to increase the number of trained health professionals as a step towards reducing maternal mortality. The focus has been on training more midwives. It would be beneficial to diversify the pool of trained health workers by including community health workers in the model to improve maternal health. Community health workers play a vital role in supporting the health of hard-to-reach populations and can be the link between pregnant women and the health care system. Programs should be developed to engage community leaders in efforts to improve maternal health in the North. These leaders can influence community members’ beliefs and behavior by encouraging the use of skilled birth attendants.

Many women in this study had no education, emphasizing the need for new strategies to improve education and school attendance in women, particularly in the North. These should include better retention strategies for teachers and incentives to encourage student attendance at primary school. Compensation for individuals who accept teaching posts in the North should be increased. There should also be on-site supervisors for schools in the North to improve staff attendance.

There is a need for alternative strategies for health promotion and education. Mass media, known for boosting uptake of interventions, is a major tool in Cameroon for health communication. However, about a third of the study population had no media exposure. Hence, alternate strategies such as text messages about maternal health to cell phones and basic health education through community health workers should be explored.

With domestic violence associated with maternal mortality, community education is needed to influence attitudes and perceptions about domestic violence at the national level. Programs that sensitize the public about the harms of domestic violence and provide safe and confidential services for victims should be developed.

The government of Cameroon needs to dedicate significant effort in the protection of young girls from early marriage and childbearing. Primarily, communities across the country must be sensitized about the adverse effects of this practice through national campaigns. The government, in collaboration with community leaders and other stakeholders, should change the laws, facilitate access to educational material, promote education of girls and increase the legal age of marriage.

### Limitations

Information on deceased women in the CDHS was limited. Only data on age and parity were available which necessitated the use of selected respondent characteristics for the deceased siblings to allow for comparisons on more determinants of maternal mortality. This also required exclusion of variables such as nutritional and health status from the analyses as these could not be ascribed to deceased siblings.

The assumption was made that deceased siblings share the same characteristics as the respondents. It is possible that some deceased siblings may have differed from their living siblings on one or more characteristics that were ascribed to them. Therefore, in light of these limitations, the findings of this study should be interpreted with caution.

Still, data provided by the siblings of deceased women contain important information and remain very useful where other data (vital registry) are limited and maternal mortality cannot be directly determined. Also, evidence suggests that living women and their deceased siblings often share similar sociodemographic attributes, supporting our approach of using respondent attributes for evaluating maternal mortality of siblings [23–25].

The modified framework included determinants of maternal mortality from the important domains of the McCarthy and Maine framework that are available in the data and cover each domain adequately. However, some domains and variables were omitted from the final model because they were unavailable or were missing for some study participants. MMRs in this study relate to a 7 year period prior to the survey date. Estimates for shorter intervals could not be computed because the direct sisterhood method is not recommended for such computations as any estimate for shorter periods are unreliable.

## Conclusion

This study measured MMRs and identified their determinants in the North and South regions of Cameroon, showing different MMRs that reinforce the need for interventions that are regionally relevant. This study has implications for programs aimed at improving maternal health and reducing maternal mortality.

## Data Availability

The 2004 and 2011 Cameroon Demographic and Health Survey datasets are publicly available at the Measure DHS program site https://dhsprogram.com/data/availabledatasets.cfm. The corresponding author can provide the formats used in this study upon request.

## Abbreviations

MDG: Millennium Development Goal
SDG: Sustainable Development Goal
MMR: Maternal Mortality Ratio
CDHS: Cameroon Demographic and Health Survey
USAID: United States Agency for International Development
OR: Odds Ratio
CI: Confidence Interval
